# Dr. Barry on the Gibraltar Fever

**Published:** 1830-04-01

**Authors:** 


XLVI.

GIBRALTAR FEVER.

On Monday evening, the 8th March, Dr. Barry read a very interesting paper on the late severe epidemic fever, which carried off more than a thousand souls, out of a population of twenty-one thousand inhabitants and sojourners on the rock of Gibraltar. The following are the prominent features of this communication, as nearly as we could collect.

1. The late Gibraltar epidemic was a fever of *one* paroxysm—cold, hot, sweating. If slight, nothing more—if severe, yellowness about the 3d day—black-vomit, hiccup—total suppression of urine, copious hæmorrhage of black blood from the nose, gums, anus, vagina, and death from 4th to 6th or 7th day, always without fever.

2. Anatomical characters—fawn-coloured mottling of the liver, as if partially boiled—black liquid in the stomach and intestines, whether the patient had vomited black or not—sepulchral yellowness of neck and breast.

3. Gibraltar is certainly one of the cleanest towns in Europe, not even excepting Bath. Well supplied with the best fresh provisions, vegetables, &c. no poor—not a single beggar.

4. The population 21,000 at the commencement of the epidemic, spread over a surface three miles long by more than one-quarter broad, besides the bay, the neutral ground, and Catalan bay village. This includes Military and all other classes in the whole territory.

5. No town, nor territory in Europe has been more improved within the last ten years.

6. Nine persons were sick of *some fever*, and two died on board a ship from the Havannah, on her passage to Gibraltar, in the Summer of 1828. Whilst this ship lay in quarantine, from 27th June to August, *three* more were ill, and one of these was received into the Civil Hospital of Gibraltar on 6th August, as "febris intermittens," but he had no paroxysm after admission.

7. The two first persons taken ill were the son and daughter of a bum-boat man, they had been on board some ship shortly before—one died on 17th the other on 20th August—both yellow, both dark vomiting. The family of the health-guard of this ship was attacked on the 21st August. A servant maid in the Civil Hospital on the 23d August. The one military case was on 2nd September. This man lodged between the two houses first attacked.

8. This disease left upwards of 5000 persons untouched who had passed it before. There is not a *single* authenticated case of second attack in Gibraltar. Of 164 military orderlies in the regimental hospitals, 141 caught the disease. Of 61 civil attendants who volunteered to attend the military sick, (being largely paid,) two only were attacked, and these were the only persons of the 61 who had not passed the disease in some former epidemic. This fact is taken from the official returns of regimental surgeons.

9. Three modes of treatment—1st, mercury with a view to salivate rapidly.—2d. free bleeding—3d. oily, and other mild aperients. The first was by far the *least* successful—the third the most so.

10. Some cases (7) of icterus appeared in 1829, from May to September. Three of these had no fever—four had passed the disease in other years.

No atmospheric or other physical phenomena different from those of the five preceding years were noticed in Gibraltar in 1828.

The impression left on our minds, from hearing the paper read, was, that, if Dr. Barry's statements are correct, (and we do not mean to attach the slightest suspicion to them,) two things out of three are proved—the contagious character of the fever — and the non-liability to second attacks. In respect to the importation of the disease from the other side of the Atlantic, we confess that we see nothing like a proof. Of the local or endemic origin of the fever we have no more doubts than we have of our own existence. But if it turns out to be authenticated, beyond all controversy, that people are not liable a second time to its attacks, that fact alone will be worth all the theories and altercations that have agitated the medical world for the last twenty or thirty years. But the advocates of importation seem not to be aware that this fact (if fully verified) will prove too much; for we believe that no old and experienced practitioner on the other side of the Atlantic will affirm that such non-liability to second attacks is a character of the fever, in the countries whence it is supposed to be imported. Indeed this character must separate it from all fevers hitherto known, excepting the eruptive fevers, and bring it back to its *old* denomination—the "*nova* pestis." We shall say no more till we hear farther from the seat of war. In our next number we shall present our readers with some important particulars respecting the medical topography of Gibraltar, from the pen of the late estimable and talented Dr. Hennen, who gives a different view of the cleanliness of that fortress.

---

